# Phase holograms for the three-dimensional patterning of unconstrained microparticles

**DOI:** 10.1038/s41598-023-35337-8

**Published:** 2023-06-06

**Authors:** Mohamed A. Ghanem, Adam D. Maxwell, Diane Dalecki, Oleg A. Sapozhnikov, Michael R. Bailey

**Affiliations:** 1 Center for Industrial and Medical Ultrasound, Applied Physics Laboratory, University of Washington, 1013 NE 40th St., Seattle, WA 98105 USA; 2 Department of Urology, School of Medicine, University of Washington, Seattle, WA 98195 USA; 3 Department of Biomedical Engineering, University of Rochester, Rochester, NY 14627 USA; 4 Physics Faculty, Moscow State University, Moscow, 119991 Russia

**Keywords:** Biomedical engineering, Acoustics, Ultrasound, Tissue engineering

## Abstract

Acoustic radiation forces can remotely manipulate particles. Forces from a standing wave field align microscale particles along the nodal or anti-nodal locations of the field to form three-dimensional (3D) patterns. These patterns can be used to form 3D microstructures for tissue engineering applications. However, standing wave generation requires more than one transducer or a reflector, which is challenging to implement in vivo. Here, a method is developed and validated to manipulate microspheres using a travelling wave from a single transducer. Diffraction theory and an iterative angular spectrum approach are employed to design phase holograms to shape the acoustic field. The field replicates a standing wave and aligns polyethylene microspheres in water, which are analogous to cells in vivo*,* at pressure nodes. Using Gor’kov potential to calculate the radiation forces on the microspheres, axial forces are minimized, and transverse forces are maximized to create stable particle patterns. Pressure fields from the phase holograms and resulting particle aggregation patterns match predictions with a feature similarity index > 0.92, where 1 is a perfect match. The resulting radiation forces are comparable to those produced from a standing wave, which suggests opportunities for in vivo implementation of cell patterning toward tissue engineering applications.

## Introduction

Remote manipulation of microparticles is important for noncontact applications including micro- and nanofabrication, lab-on-chip technologies, and tissue engineering. Tissue engineering provides an alternative approach to replace injured or disease organs or tissue^1,2^. Spatial patterning of cells into microstructures to form two- and three-dimensional (2/3D) assemblies is essential for providing a shape or structure for complex tissue regeneration^3–5^. 3D structural cellular arrangements have higher success in engineered tissue applications^6–8^. Several methods have been used to pattern cells in vitro using polymer matrix templates^9^ and bioprinting^10^. These methods have their advantages and drawbacks. Bioprinting can achieve complex desired shapes, but the cellular structure is constructed in a point-by-point manner, a time consuming approach that requires a complex setup^10^. Meanwhile, matrix-based methods pattern molds by changing the matrix properties, which is a more rapid method but is not suitable for complex shapes^11,12^. An alternative method is acoustic manipulation which is capable of remotely arranging a large number of cells simultaneously without direct physical contact with the cells^13,14^. Cells exposed to an acoustic field scatter the field leading to an acoustic radiation force that can spatially reposition the cell^15^.

Acoustic radiation forces have been applied for a wide variety of remote manipulation applications, such as microbubbles^16^ or solid objects in vivo^17^, or selective single cell^18^ or small particle manipulation for in vitro research^19^. The use of radiation forces to move a mass of particles form 3D structures is of particular interest. Most rapid and non-invasive 3D alignment of microstructures have utilized a standing wave^8,20–22^ generated by a transducer and a reflector, or multiple transducers facing each other. This setup forms alternating planes of zero and high pressure amplitude, known as nodes and antinodes, that are perpendicular to the propagation direction and are spaced at half-wavelength intervals. Acoustic radiation forces imparted by a standing wave direct microparticles or cells towards the nodes or antinodes of the field depending on their acoustic properties relative to those of the surrounding medium^23^. For tissue engineering applications, the 3D microstructure can be held in position using photopolymers^24,25^ or a hydrogel medium^26–30^. This acoustic patterning technology holds promise as a tool for tissue engineering.

An important goal of tissue engineering is to assemble cellular structures in vivo directly. It was previously shown that a two-transducer system can generate a standing wave where their beams crossed to pattern endothelial cells forming a complex 3D microvessel network in a hydrogel volume in vitro^3,27^ and in vivo^31^ site-specifically and non-invasively. However, standing waves are difficult to generate in some regions of the body because of limits on where transducers can be placed on the body to transmit sound without obstruction. Conversely, a traveling wave cannot hold a stable pattern and pushes the particles away in the direction of propagation. In this work, we utilized phase holograms that can produce complex pressure fields from one transducer^32^ to create a 3D standing wave pattern similar to the one used to form a 3D microvessel network^31,33,34^. Previous work has utilized phase holograms to achieve complex 2D particle patterning with radiation forces^35^ and acoustic streaming^36,37^, while relying on boundaries to mitigate the radiation forces from a single transducer from pushing the particles away. However, these conditions cannot be generated in most in vivo scenarios.

**Figure 1:**
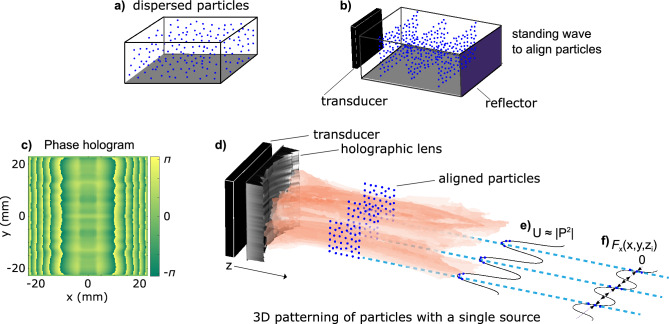
Acoustic radiation forces align particles along parallel planes of pressure. Particles are dispersed in a container (**a**), and are aligned in a standing wave field using a transducer and a reflector (**b**). Our work uses a phase hologram (**c**) to fabricate a holographic lens coupled with a transducer to create pressure planes that are parallel to the wave propagational axis (**d**). The pressure planes have no axial pressure gradient near the region of interest *z*_i_ where the Gor’kov potential is directly proportional pressure amplitude for particles with density similar to the surrounding medium (**e**) and the resulting forces aligns the polystyrene microspheres along the nodal pressure regions (**f**).

Here, we used a holographic lens with one transducer to assemble suspended microspheres using acoustic radiation forces only along a predefined 3D pattern in vivo-mimicking environment while the spheres experience no pushing acoustic forces. Our goal was to use a single transducer to create an acoustic field of parallel planes of pressure nodes and antinodes (Fig. 1a,b) to suspend cell-mimicking particles in this pattern over several wavelengths in the axial distance. Such a field is similar to a standing wave, but the planes lay parallel to and with their normal orthogonal to the acoustic axis. For particles much smaller than the acoustic wavelength, the radiation forces are predicted by the Gor’kov potential^23^, where the forces are proportional to the gradients of acoustic pressure and velocity, and the relative properties of the particle and medium. Particles that are neutrally buoyant—cells in water are nearly so—eliminate the velocity gradient contributions to the radiation forces. Thus, the radiation forces and particle alignment are dependent on the pressure gradients alone. We designed phase holograms using an analytical method and the iterative angular spectrum approach (IASA)^32^ to fabricate lenses that produce parallel pressure planes with zero pressure gradient in the axial direction over a designated region at a specified distance from the transducer. Therefore, the radiation forces align neutrally buoyant cell-mimicking microspheres in water along the desired parallel plane distribution with no axial motion present in a cuvette to mimic conditions for in vivo implementation, see Fig. 1c–f.

## Results and discussion

**Figure 2:**
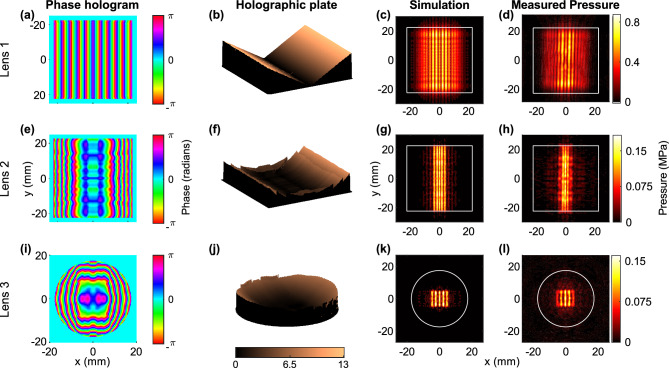
Results of holographic acoustic fields. Each row represents the result from lens 1 (top), 2 (middle) and 3 (bottom). The columns are phase holograms (**a**,**e**,**i**) used to fabricate the lenses (**b**,**f**,**j**), simulation of desired image of pressure amplitude in the transverse *xy*-plane (**c**,**g**,**k**), and holography scan measurement of the acoustic fields at the desired image plane in the transverse *xy*-plane (**d**,**h**,**l**). In the acoustic pressure fields subplots, the solid white border outlines the piezoceramic active acoustic element. The field produced from lens 1 is equivalent to that of two sources with their acoustic axes separated by an angle 2*θ*_w_ (**b**) which outputs approximately a standing wave in the transverse direction (**c**,**d**). Lenses 2 and 3 produced five parallel axial planes as predicted by simulation (**h**,**l**). For lens 2, the outer planes are weakly formed and never achieve full illumination (**h**), while lens 3 produced five planes with outer planes slightly weaker than predicted by simulation (**g**,**h**).

The results were produced separately for three different custom-made transducers, each with a holographic lens to synthesize a unique pressure field. Lenses 1 and 2 were attached to 1.5-MHz, 45-mm square piezoceramic elements while lens 3 was attached to a 2-MHz, 35-mm circular piezoceramic element (see Supplementary Information Fig. S3). Lens 1 was composed of two congruent rectangles angled toward each other by *θ* = 30˚ from the horizontal (Fig. 2a,b) with the acoustic rays entering the water at an angle of entry *θ*_w_ = 13° to form two plane waves intersecting at 2*θ*_w_. This lens generated a forward propagating wave, with a standing wave component in the transverse dimension that produced parallel planes of pressure separated by *d*_o_ = λ/[2 × sin (*θ* − *θ*_w_)] = 2.22 mm where *λ* is the acoustic wavelength (see “Methods”).

The pressure field from lenses 2 and 3 (Fig. 2g,k) were generated by using the phase boundary condition obtained from IASA (Fig. 2e,i) to produce the desired pressure image at 46 and 35 mm from the source. IASA iterates over the phase boundary condition until convergence is reached to satisfy a target pressure image at the specified location^32^ (see “Methods”). A target location that is too close to, or too far from, the source and the phase hologram cannot synthesize and maintain uniform parallel pressure planes over a distance. Therefore, the imposed target pressure image location was placed in the translational region of the field, near the end of the Fresnel region and before the Fraunhofer diffraction region. This region allowed the target image to be at a distance where the development of spherical spreading can maintain the shaped image for 2–3*λ* axial distance. For sources with the effective radius much larger than the wavelength, the translational region starts prior to the last on-axis pressure amplitude maximum^38^(see “Methods”). The phase boundary condition^39^ was unwrapped to achieve a continuously smooth morphology of the fabricated lens surface (see Supplementary Information).

### Holographic acoustic field

Acoustic holography^40^ was performed to scan the pressure fields produced by the transducers coupled with the holographic lenses submerged in water. The field was scanned in a plane orthogonal to the acoustic axis by a 200-μm diameter capsule hydrophone (HGL-0200, Onda Corp., CA, USA) that recorded the waveform in a square grid of points at a maximum of *λ*/2 grid spacing for all transducers^40^. The recorded waveforms were used to find the angular spectrum of the holographic lens and construct the source vibration and the 3D complex pressure and particle velocity field (see “Methods”).

Figure 2 shows the source phase, holographic lens, and the pressure amplitude measured and simulated at the desired target distance from the source. The average spacing between parallel planes from lens 1 was measured to be *d*_o_ = 2.26 ± 0.027 mm with an error of 2.15 ± 1.22%. Using this value of *d*_o_, *θ*_w_ was calculated to be 12.75° ± 0.17°. The feature similarity index (FSI)^41^ provides a value (0–1) on the agreement between measured and simulated patterns with 1 being perfect agreement. Pressure measurements for lens 1, 2, and 3 achieved an FSI of 0.950, 0.939, and 0.953 respectively. The pattern of parallel planes produced from lens 1 extended from 20 to 45 mm in the axial direction (Supplementary Information Fig. S4), while the desired pressure images from lens 2 and 3 were formed at 48.0 and 35.6 mm from the source. The difference between the imposed and measured pressure locations was attributed to the mismatch between the sound speed in water and lens material which was not accounted for in the simulation.

Measurement of the pressure field from lens 2 (Fig. 2f) showed the formation of three distinct planes, with the outer planes having lower intensity than the center. The three planes extended for 2.5 mm (2.5*λ*) with equal and uniform pressure level over the central 1.5 mm axial extent while the 0.5 mm pre and post central region had higher variance between the planes’ intensity levels (see Fig. S4). Similarly, lens 3 formed five parallel axial planes extending over 1.5 mm (2*λ*). The outer planes had a relatively lower intensity level than was predicted by simulation (Fig. 2l and see Supplementary Information).

### Particle alignment

**Figure 3:**
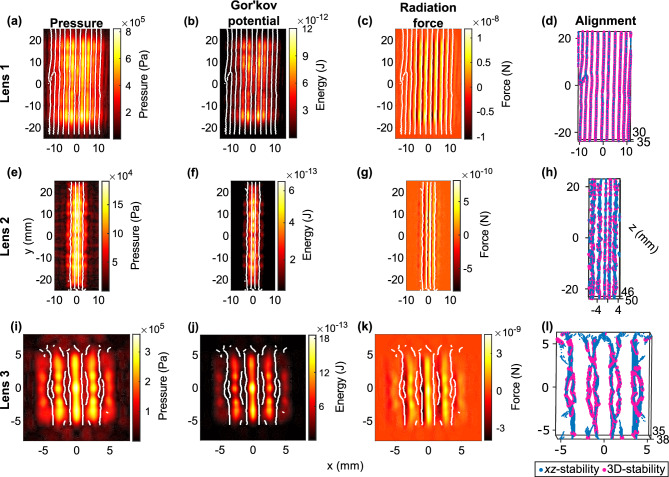
Acoustic radiation forces imparted on polyethylene microspheres that are 75–90 μm in diameter under acoustic pressure values obtained in Fig. 2. The direction of the force in the *xy*-plane for all lenses (**a**,**e**,**i**) aligns the spheres in pressure nodes. Zero force locations in the *xy*-plane are where the potential energy is at minimum (**b**,**f**,**j**) as marked by the white lines indicating particle agglomeration positions. The alignment position of particles with three-dimensional stability are shown for all lenses (**d**,**h**,**l**) where saddle points with *xz*-stability (violet dots) are surrounded by fully stable positions (pink dots). Lens 3 had additional local minima where spheres aligned along shorter vertical planes between the main stability planes (**i**–**l**).

Gor’kov potential was constructed from the measured pressure and velocity fields to predict the acoustic radiation forces on polyethylene microspheres (75–90 μm diameter) along different spatial positions (see Methods). The radiation force aligned the microspheres in the nodal planes, which are marked by white lines in Fig. 3. In the *xy* transverse plane, the force in the *x*-direction was zero at the nodes and antinodes (Fig. 3c,g,k). The potential’s local minima coincided with the pressure nodes where the radiation forces were stable and spheres were directed toward the nodes (Fig. 3b,f,j). The microspheres aligned and formed a minimum of 6, 4 and 4 vertical lines in the *xy*-plane from the radiation force imparted by lens 1, 2 and 3, respectively. The pressure field produced from lens 3 contained regions of slightly lower pressure than the surrounding pressure within an antinode, thus creating secondary potential energy minima where trapping occurred as indicated by short white lines in Fig. 3i–l. The stable particle distribution was predicted from the local minima of Gor’kov’s potential^42^ as shown in Fig. 3d,h,l. Lenses 2 and 3 exhibited potential energy saddle points^42^ along the *y*-dimension where a small perturbation could cause microspheres to become unstable and move in *y*. However, these points had two-dimensional *xz* stability and were surrounded by 3D stable regions, which stabilized the particles in those saddles (Fig. 3d,h,l).

**Figure 4:**
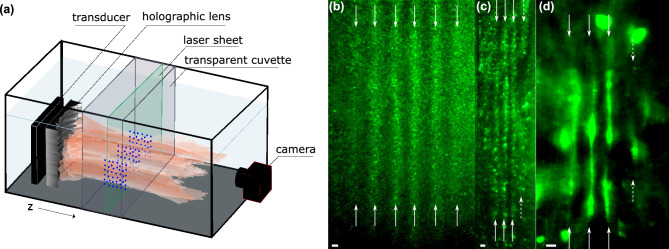
Setup of acoustic radiation force alignment of polyethylene microspheres in water (**a**). Plots (**b**–**d**) capture images of alignment where white arrows mark the location of nodal pressure planes. Lens 1 strongly aligns microspheres along six vertical lines between the anti-pressure nodes (**b**). Lens 2 forms two vertical lines of microspheres between three anti-nodal vertical pressure planes with slightly weaker alignment on the outside left and the weakest alignment plane is on the outside right marked by dashed white arrows (**c**). Similarly, lens 3 forms 5 anti-nodal pressure planes with 4 vertical trapping regions in between, with the weakest trapping located on the outside right and marked by a white dashed arrow (**d**). The scale bar at the bottom of each subplot (**b**–**d**) is 1 mm in length. A high concentration of polyethylene microspheres was used to visualize the acoustic radiation force alignment and secondary trapping regions from lens 3 by pushing the microspheres from the anti-nodal planes forming a negative image in Fig. S7, and Movie S4.

To validate predictions, we aligned microspheres in a custom-made cuvette made from an acoustically transparent membrane material (Fig. 4). Transducers were submerged in a degassed water tank with the cuvette. A few drops of microspheres solution (0.032 g/mL) were added inside the cuvette (see “Methods”). The cuvette dimension along the axial direction was approximately 1 cm. A 1.2-mm thick laser sheet illuminated the transverse plane of interest to photograph and record the microsphere suspension and patterning. The maximum pressure produced for the experiment was 0.5, 0.6 and 1.7 MPa for lenses 1, 2 and 3 respectively. No axial motion in the region of interest was observed. Figure 4 shows the alignment of microspheres between antinodes for all lenses. All lenses patterned microspheres along the nodes as predicted and shown in Fig. 3, with the outer lines having regions with low concentration of particles due to the weaker acoustic intensity, thus weaker forces. The horizontal spacings between the lines for lens 1–3 from the holography-scan-based force measurements in Fig. 3d,h,l are 2.34 ± 0.17, 1.75 ± 0.08, and 1.85 ± 0.15 mm, while from Fig. 4, the spacings were measured to be 2.37 ± 0.32, 1.57 ± 0.18, and 1.35 ± 0.14 mm, respectively. Supplementary Movies S1–S3 show alignment of particles from the acoustic exposure of each lens. The alignment of spheres in the secondary trapping regions produced from lens 3 was observed using a high concentration of microsphere solution to show the antinodes by a complete absence of microspheres (see Supplementary Figs. S7; Movie S4).

**Figure 5:**
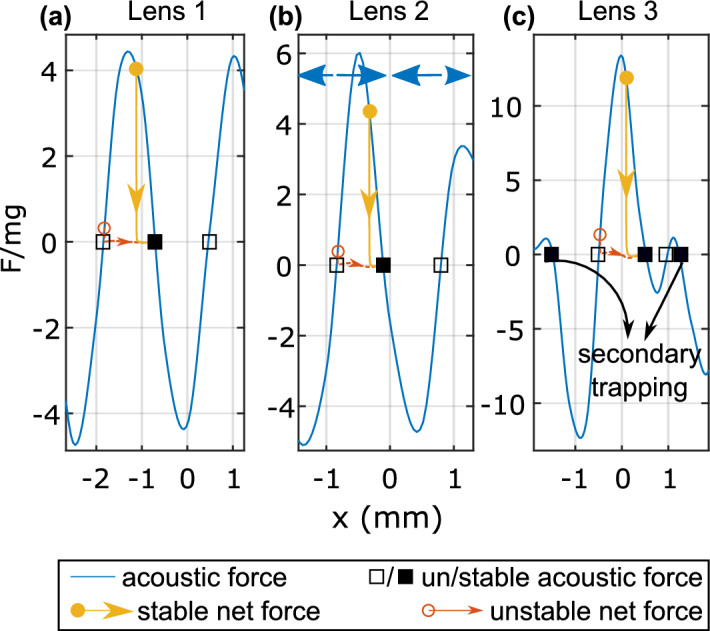
Normalized forces experienced by a single polyethylene microsphere located at the target pressure image location at different starting *x* positions for lenses 1 to 3 (**a**–**c**). The forces are normalized by the microsphere’s weight in a vacuum. The solid curve is the acoustic radiation force, lines with arrows are net force when the initial position is at stable (solid circle) or unstable (open circle) region before the acoustic exposure. Locations with zero *x*-component radiation acoustic force are marked by square that show stable (solid) or unstable (open) equilibrium.

The net force due to the radiation and hydrodynamic forces was calculated to show the trajectory of a single microsphere placed in water on the *x*-axis at the target pressure plane location with a maximum pressure amplitude equal to 1 MPa (see “Methods”). The trajectory of microsphere from different initial positions along *x* in the acoustic field was plotted in Fig. 5. Microspheres placed near stable positions translated toward a nodal location, while those placed near unstable positions close to an antinode were pushed away and floated with a negligible inertia before eventually reaching a stable position. The net restoring force acting on a microsphere could reach up to tenfold its weight, with stronger focusing of higher frequency producing the largest radiation force, as shown by lens 3.

We used lenses to shape travelling waves and align microspheres along parallel pressure planes within a limited 3D space at specified locations. Lens 1 produced parallel planes over an extended distance of approximately 25*λ* in the nearfield, similar to a non-diffracting Bessel beam^43^ that can be experimentally created by an axicon^44^. Both lens 1 and Bessel beams share similar boundary conditions. A Bessel beam has an axisymmetric vibrational amplitude and a characteristic angle, while lens 1 has a uniform vibrational amplitude symmetric about the *y*-axis and an entry angle (see derivation in Supplementary Information). Holographic lenses 2 and 3 created parallel pressure planes that extended for only 2–3*λ* but allowed for precision in designing 3D pressure fields and complex patterns. However, the field shaping was confined to a nearfield region of the source before the spreading of the acoustic beam, which occurs proximal to the Rayleigh distance defined as the source area over the wavelength^45^. The distance of manipulation is constrained by the source size and frequency, while the highest pressure pattern resolution is limited to *λ*/2. Sensitivity analysis of the source boundary conditions (see Supplementary Information) showed greater dependence on the phase than the amplitude boundary condition for higher pressure field accuracy. Phase unwrapping produced the most accurate phase boundary condition but resulted in higher attenuation, causing weaker alignment forces of the outer planes. Therefore, the surface morphology chosen for the lens was critical for accuracy.

The particle patterning matched our theoretical prediction and achieved suspension of particles without motion in the axial direction as intended. We calculated radiation forces from maximum acoustic pressure of 1 MPa on mouse embryonic myofibroblasts cells^34^ (with density *ρ* = 1.05 g/cm^3^, sound speed *c* = 1529 m/s, and *r* = 3 μm) from lenses 1–3 to be 2.33 ± 0.350, 4.51 ± 3.16, and 4.62 ± 1.21 piconewtons (pN). These values are comparable to those shown in Ref.^34^ that reached 2.2 pN from a standing wave pattern at an amplitude of 0.2 MPa. These results demonstrate the feasibility of in vivo cell patterning using single transducer methods.

## Conclusion

We designed and fabricated holographic lenses to reshape the pressure field and produce pressure planes that were parallel to the acoustic axis and mimicked standing wave behavior over a limited region remote from the transducer surface. The pressure field generated acoustic radiation forces that manipulated subwavelength particles and steered them to the nodes while avoiding undesirable axial motion. Calculations showed that the forces produced by the propagating wave were equivalent to those produced by a standing wave, with the forces up to tenfold greater than the particle weight. This technology has the potential for controlled cellular patterning for in vivo vascularization and other tissue engineering applications.

## Methods

### Holographic lens design

Lens 1 was designed using two angled congruent sides facing each other with an angle equal to 2*θ* to produce a pressure in three dimensions defined by:

1 $$p\left(x,y,z\right)=\frac{1}{4{\pi }^{2}}{\iint }_{{k}_{x}^{2}+{k}_{y}^{2}\le {k}^{2}}S({k}_{x},{k}_{y}){\mathrm{e}}^{i{k}_{x}x+i{k}_{y}y+i\sqrt{{k}^{2}-{k}_{x}^{2}-{k}_{y}^{2}} z}d{k}_{x}d{k}_{y}$$

where *k* = 2*π*/*λ*, *k*_x_, *k*_y_, and *k*_z_ are the *x, y* and *z* wavenumbers, respectively. And

2 $$S({k}_{x},{k}_{y})=\frac{\rho c{v}_{o}{L}_{x}{L}_{y}}{2\sqrt{1-\frac{{k}_{x}^{2}+{k}_{y}^{2}}{{k}^{2}}}}\left\{{\mathrm{e}}^{-\frac{i{k}_{1}{L}_{x}}{4}}\mathrm{sinc}\left(\frac{{k}_{1}{L}_{x}}{4}\right)+ {\mathrm{e}}^{\frac{i{k}_{2}{L}_{x}}{4}}\mathrm{sinc}\left(\frac{{k}_{2}{L}_{x}}{4}\right)\right\}\times \mathrm{sinc}\left(\frac{{k}_{y}{L}_{y}}{2}\right)$$

where ${k}_{1}= {k}_{x}\mathrm{cos}{\theta }_{w}+\sqrt{{k}^{2}-{k}_{x}^{2}-{k}_{y}^{2}}\mathrm{sin}{\theta }_{w}$, ${k}_{2}= {k}_{x}\mathrm{cos}{\theta }_{w}-\sqrt{{k}^{2}-{k}_{x}^{2}-{k}_{y}^{2}}\mathrm{sin}{\theta }_{w}$, *L*_x_ and *L*_y_ are the *x* and *y* dimensions of the full rectangular source, *υ*_o_ is the velocity boundary condition, and *θ*_w_ is the angle of entry of acoustic rays into the water medium defined from Snell’s law, see Supplementary Information for full derivation of Eq. (2).

IASA^32^ was used to design lens 2 and 3 by iterating over the source phase boundary condition to reach a target pressure image imposed at a specific axial distance from the source. To determine the location of the target, the Rayleigh integral^45^ was used to simulate the pressure amplitude distribution on the acoustic axis. The last pressure amplitude maximum was then chosen as the desired distance. The imposed pressure target for each lens was a binary image of five vertical lines, each 10 mm in height and equidistant from each other. The imposed pressure targets for each lens are shown in Supplementary Information Fig. S2. The termination of the iteration was defined to be when the difference between the second norm of the phase boundary condition from two consecutive iteration steps to be ${\Vert {\phi }_{i}-{\phi }_{i-1}\Vert }_{2}$≤ 1e−3.

### Acoustic holography

A continuous wave (CW) analysis^40^ of the hydrophone scan at the plane *z* = *z*_p_ was used to record the acoustic hologram of the source and to construct the complex pressure in 3D (see Supplementary Information). The angular spectrum at the scan plane *z*_p_ was defined as such:

$$S\left({k}_{x},{k}_{y}\right)=\underset{-\infty }{\overset{\infty }{\int }}\underset{-\infty }{\overset{\infty }{\int }}p\left(x,y\right) {e}^{-i{k}_{x}x-i{k}_{y}y}dxdy,$$

which was used to construct the complex pressure amplitude at a plane normal to the acoustic axis located at *z*_i_:

3 $$p\left(x,y,{z}_{i}\right)=\frac{1}{4{\pi }^{2}}\iint S\left({k}_{x},{k}_{y}\right){e}^{i{k}_{x}x+i{k}_{y}y+i\sqrt{{k}^{2}-{k}_{x}^{2}-{k}_{y}^{2}} ({z}_{i}-{z}_{p})} d{k}_{x}{dk}_{y}$$

For a harmonic wave, the particle velocity complex amplitude is expressed from the complex amplitude of the acoustic pressure to be:

$$\overrightarrow{v}=\frac{\nabla P}{i\omega \rho }$$

Therefore, the *j*th component of the velocity vector from Eq. (3) is:

4 $${v}_{j}\left(x,y,{z}_{i}\right) =\frac{1}{4{\pi }^{2}c\rho }\iint \frac{{k}_{j}}{k}S\left({k}_{x},{k}_{y}\right){e}^{i{k}_{x}x+i{k}_{y}y+i{k}_{z}({z}_{i}-{z}_{p})} d{k}_{x}{dk}_{y}.$$

*Acoustic radiation forces* on the microspheres are calculated from Gor’kov potential since the spheres are much smaller than the acoustic wavelength (r/*λ* ≈ 0.1). For a harmonic burst, the radiation force on a particle is defined as:

5 $${F}_{i}=\frac{\pi {r}^{3}}{\rho {c}^{2}}\mathrm{Re}\left[{-\frac{2{f}_{1}}{3}P}^{*}\frac{\partial P}{\partial {x}_{i}}+\frac{{c}^{2}{f}_{2}}{{\omega }^{2}}\frac{\partial {P}^{*}}{\partial {x}_{j}}\frac{{\partial }^{2}P}{\partial {x}_{i}\partial {x}_{j}}\right]$$

where the Einstein summation notation is used (see Supplementary Information for derivation). The force is defined as $\overrightarrow{F}=-\nabla U$ where Gor’kov potential *U*^23^ is:

$$U=\pi {r}^{3}\rho \left[\frac{{\left|P\right|}^{2}}{3{\rho }^{2}{c}^{2}}{f}_{1}-\frac{{\left|\overrightarrow{v}\right| }^{2}}{2}{f}_{2}\right]$$

And $$f_{1} = 1 - \frac{{{\text{c}}^{2} \rho }}{{c_{l}^{2} \rho _{*} }}\frac{1}{{1 - 4c_{t}^{2} /3c_{l}^{2} \$ }}$$ and $${f}_{2}=\frac{2\left({\rho }_{*}-\rho \right)}{(2{\rho }_{*}+ \rho )}$$^46^.

The radius of the microsphere is *r*, and the longitudinal and transverse sound speeds, and density are *c*_*l*_ (2566 m/s), *c*_*t*_ (1273 m/s) and ρ_*_ (0.922 g/cm^3^)^47^, while *ρ* (1 g/cm^3^) and *c* (1500 m/s) are those of the surrounding water, *P* and $\overrightarrow{v}$ are the incident acoustic pressure and particle velocity complex amplitudes.

### Alignment of microspheres

Approximately 0.3 g of polyethylene microspheres were placed in a glass bottle with few drops of liquid detergent added as a surfactant, then 125 mL of deionized and degassed water was added to the bottle. A magnetic bar was inserted into the bottle, and it was continuously stirred by a magnetic mixer throughout the experiments. Microspheres were placed few drops at a time in a cuvette 3D printed from polylactic acid (PLA) filament (Ultimaker, Framingham, MA) with acoustically transparent side walls made from 12.7-μm, polyester, clear film (McMaster-CARR, Elmhurst, IL). All transducers were operated in a pulsed mode, transmitting 100-cycle pulses at 10% duty cycle. Microspheres aligned along the nodal planes created scattering of the light and were visible. A camera was placed outside the tank facing the transducer to capture the alignment. The alignment images presented in Fig. 4 used a brightness threshold as cutoff to improve the contrast of regions where microspheres agglomerated.

### Force analysis

A polyethylene microsphere placed in the acoustic field will experience radiation forces imparted by the acoustic field and hydrodynamic drag by fluid due to the microsphere’s motion relative to the surrounding fluid. The sphere will undergo Stokes’ drag, virtual mass, and Basset–Boussinesq history forces^48^. Generally the inertial forces are minimal and only Stokes’ drag is important^49^. However, we calculated the added mass since the microspheres have a density similar to the surrounding water, and accounted for Basset–Boussinesq history force to account for the unsteady flow due to the initial sudden acceleration of the sphere, which has been shown to affect the early time of motion^50^. It is assumed that fluid streaming from acoustic exposure is minimal due to the presence of the cuvette walls^51^. Thus, only the sphere’s motion contributes to the drag forces. The total drag forces on the sphere are defined as follow:

6 $$F\left(t\right)=6\pi \mu r\frac{dx}{dt}+\frac{2}{3}\rho {\pi r}^{3}\frac{{d}^{2}x}{d{t}^{2}}+6\pi \mu r{K}_{\mu }\left(t\right)\frac{dx}{dt}$$

where the terms from left to right are defined as Stokes’ drag, added mass, and Basset forces^52^. The position of the sphere is *x*, ${K}_{\mu }\left(t\right)=1/\sqrt{\mu \pi t/\rho {r}^{2}}$ is the memory kernel of the Basset term for a rigid sphere with its relative viscosity to water is infinite^48,52^, *μ* = 1.016 × 1e−3 Pa·s is the dynamic viscosity of water. The net force ***F***_net_ experienced by the microsphere is given by:

7 $${{\varvec{F}}}_{\mathrm{net}}\left(x,t\right)=m\frac{{d}^{2}{\varvec{x}}}{d{t}^{2}}={{\varvec{F}}}_{a}\left(x\right)+{\varvec{F}}\left(t\right)$$

where *m* is the mass of the polyethylene microsphere.

The order of Eq. (7) is reduced and rewritten as a system of first order differential equations to solve for a single microsphere’s trajectory using Runge–Kutta iterative method^53^ with MATLAB^®^ built-in solver.

It is important to mention that ***F***_net_ in Eq. (7) is for a single microsphere and the radiation force term ***F***_*a*_ does not take into consideration the scattering from neighboring particles^30^. The radiation force calculation in Eq. (5) assumes an inviscid fluid because the viscous fluid layer or acoustic boundary layer surrounding the particle is much less than the particle radius^54^. The maximum acoustic boundary layer is $\delta =\sqrt{2\mu /\rho \omega }=0.5, \mathrm{ \mu m}$, while the microsphere diameter range is 75–90 μm. Furthermore, fluid viscosity is the only opposing force to the radiation force, thus the assembly speed is dependent on the fluid viscosity. In an inviscid fluid, a microsphere placed slightly off from an acoustically stable equilibrium position will oscillate about the position as in an undamped mass-spring system.

## Supplementary Information

Supplementary Information 1.

Supplementary Video 1.

Supplementary Video 2.

Supplementary Video 3.

Supplementary Video 4.

## Data Availability

The data that support the findings of this study are available in the Supplementary information files.
