# Supplementary material for: Phase holograms for the three-dimensional patterning of unconstrained microparticles
Source: Sci Rep. 2023 Jun 6;13:9160. doi: 10.1038/s41598-023-35337-8 (PMC10244404; doi:10.1038/s41598-023-35337-8)
Supplement: Supplementary file 1 — Supplementary Information 1. [file 41598_2023_35337_MOESM1_ESM.docx]

Supplementary Information

# Phase holograms for the three-dimensional patterning of unconstrained microparticles

*Mohamed A. Ghanem,***Adam D. Maxwell, Diane Dalecki, Oleg A. Sapozhnikov, and Michael R. Bailey*

# Design of holographic lenses

## Phase hologram 1

Holographic lens 1 is a square element that is bisected through the middle where each half is angle by *θ* from the horizontal, so that the total angle between both halves is 2*θ*. An acoustic ray emitted by the piezoceramic will propagate though the Lens material with sound speed *c*_1_ and enter the water at an angle *θ*_w­_ to propagate with the sound speed of water *c*. The separation between planes is calculated from standing wave separation of a source an reflector which is equal to *λ*/2; however, due to the angling of the sources and Snell’s law correction between the water and lens material, the separation is *d*_0_ = λ/[2×sin (*θ*-*θ*_w_)]. Figure S1a shows the geometric configuration of lens 1.


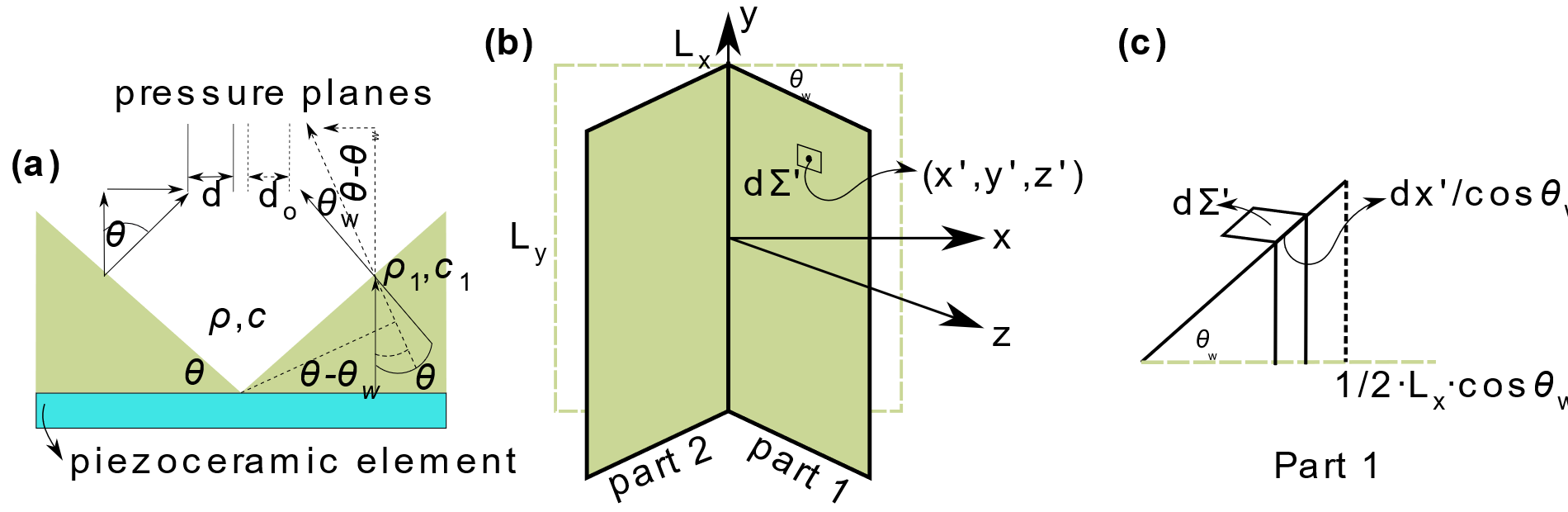


Figure S1. Design of holographic lens 1. Geometric configuration of the ray acoustic emission from the piezoceramic element and its propagation through the lens material and entry to water in order to form parallel pressure planes in water with separation do (a). Coordinate system used to derive the closed form solution for lens 1 (b).

The angular spectrum of a vibrating source can be expressed as ^1^:

| $S\left( k_{x},k_{y} \right)=\frac{\rho c}{\sqrt{1-\frac{k_{x}^{2}+k_{y}^{2}}{k^{2}}}}\iint v\left( \vec{r^{'}} \right)e^{-i\left( k_{x}x^{'}+k_{y}y^{'}+\sqrt{k^{2}-k_{x}^{2}-k_{y}^{2}} z^{'} \right)} d\Sigma^{'}$ | (S1) |
| --- | --- |

where $\Sigma$ is the source surface, $v\left( \vec{r^{'}} \right)$ is the normal velocity amplitude, $\vec{r^{'}}=(x^{'},y^{'},z^{'})$is a point on the surface, and $d\Sigma^{'}$is the surface element. In our case, for a piston, $v\left( \vec{r^{'}} \right)=v_{o}$ is a constant, and $d\Sigma^{'}=dx^{'}dy^{'}/\cos\theta_{w}$. Also, note that ${z^{'}=x}^{'}\tan\theta_{w}$. Integration of equation (S1) for part 1 of the lens yields:

$$S_{1}\left( k_{x},k_{y} \right)=\frac{\rho cv_{o}}{\cos\theta_{w}\sqrt{1-\frac{k_{x}^{2}+k_{y}^{2}}{k^{2}}}}\int_{0}^{\frac{L_{x}\cos\theta_{w}}{4}} e^{-i\left( k_{x}+\tan\theta_{w}\sqrt{k^{2}-k_{x}^{2}-k_{y}^{2}} \right)x^{'}}dx^{'}\int_{-\frac{L_{y}}{2}}^{\frac{L_{y}}{2}} e^{-ik_{y}y^{'}} dy^{'}$$

We know that $\sin\gamma=\frac{e^{i\gamma}-e^{-i\gamma}}{2i}$ and $\mathrm{sinc} \gamma=\frac{\sin\gamma}{\gamma}$. Therefore, the angular spectrum from part 1 becomes:

| $S_{1}(k_{x},k_{y})=\frac{\rho cv_{o}L_{x}L_{y}}{2\sqrt{1-\frac{k_{x}^{2}+k_{y}^{2}}{k^{2}}}}\left\{ e^{-\frac{ik_{1}L_{x}}{4}}\mathrm{sinc}\left( \frac{k_{1}L_{x}}{4} \right)\times sinc\left( \frac{k_{y}L_{y}}{2} \right) \right\}$ | (S2) |
| --- | --- |

where $k_{1}= k_{x}\cos\theta_{w}+\sqrt{k^{2}-k_{x}^{2}-k_{y}^{2}}\sin\theta_{w}$.

For part 2, ${z^{'}=-x}^{'}\tan\theta_{w}$, and *x’* integration limits are $-{\frac{L_{x}\cos\theta_{w}}{4}\leq x}^{'}\leq0$ to give the following angular spectrum:

| $S_{2}(k_{x},k_{y})=\frac{\rho cv_{o}L_{x}L_{y}}{2\sqrt{1-\frac{k_{x}^{2}+k_{y}^{2}}{k^{2}}}}\left\{ e^{\frac{ik_{2}L_{x}}{4}}\mathrm{sinc}\left( \frac{k_{2}L_{x}}{4} \right)\times sinc\left( \frac{k_{y}L_{y}}{2} \right) \right\}$ | (S3) |
| --- | --- |

where $k_{2}= k_{x}\cos\theta_{w}-\sqrt{k^{2}-k_{x}^{2}-k_{y}^{2}}\sin\theta_{w}$. The total angular spectrum is the summation of equation (S2) and (S3).

$$S(k_{x},k_{y})=\frac{\rho cv_{o}L_{x}L_{y}}{2\sqrt{1-\frac{k_{x}^{2}+k_{y}^{2}}{k^{2}}}}\left\{ e^{-\frac{ik_{1}L_{x}}{4}}\mathrm{sinc}\left( \frac{k_{1}L_{x}}{4} \right)+ e^{\frac{ik_{2}L_{x}}{4}}\mathrm{sinc}\left( \frac{k_{2}L_{x}}{4} \right) \right\}\times sinc\left( \frac{k_{y}L_{y}}{2} \right)$$

## Phase hologram 2 and 3

The lenses were designed using iterative angular spectrum approach *IASA* ^2^. Figure S2 shows the desired binary image imposed to achieve the desired pressure output, the source phase needed, and the phase lenses resulting from phase wrapping ^3^.


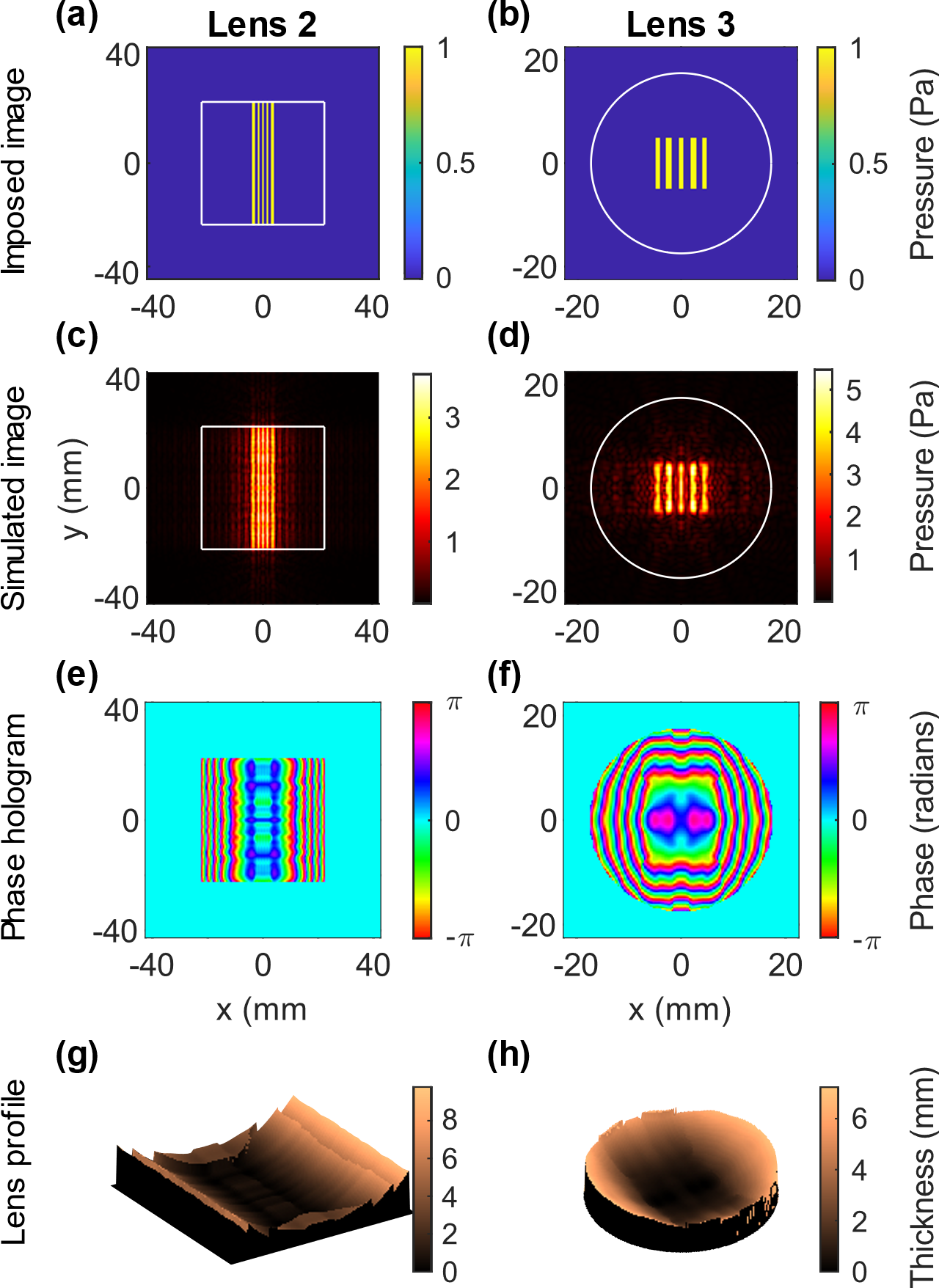


Figure S2. Iterative angular spectrum approach setup and results for holographic lenses 2 and 3. The imposed pressure image for lens 2(a) and 3(b) were chosen to be located at 46 and 35 mm, respectively. Pressure results from simulation is shown as a multiple of a 1Pa source amplitude (c and d) with the source phase (e and f) to fabricate the lenses (g and h).

# Holographic lenses and transducers fabrication

The holographic lenses were 3D printed from various photopolymer material. Lens 1 was made from a rigid transparent photopolymer, VeroClear (Stratasys, Valencia, CA) with *ρ* = 1.2 g/cm^3^ and *c* = 2560 m/s. Lens 2 was 3D printed from Accura 60 (3D Systems Inc., Rock Hill, SC) with *ρ* = 1.21 g/cm^3^ and *c* = 2570 m/s and lens 3 from Somos PerForm (Covestro, Pittsburgh, PA) with *ρ* = 1.65 g/cm^3^ and *c* = 3278.4 m/s.

The transducer cases were 3D printed to have the lens sit against the piezoelectric element. A ¼ wavelength thick matching layer with c = 2980 m/s and *ρ* = 2.88 g/cm^3^ was placed between the piezoelectric element and the holographic lens for efficient acoustic transmission for transducers 1 and 2. The matching layer was prepared from Al_2_O_3_ powder and Pro-Set infusion epoxy (Pro-Set Inc, Bay City, MI) with 2.45 to 1 weight mix ratio of INF-114 resin and INF-212 hardener, respectively. The mix was stirred until it became uniform, then it was degassed to release air bubbles. The paste was placed onto the piezoelectric elements, then the lens was pressed against it and left to cure for 24 hours, Figure S3. Transducer 3 had the piezoelectric elements pressed against the lens directly and attached using the same Pro-Set infusion epoxy without matching due to the higher impedance of the lens material. All transducers were electrically matched to 50 Ohms.


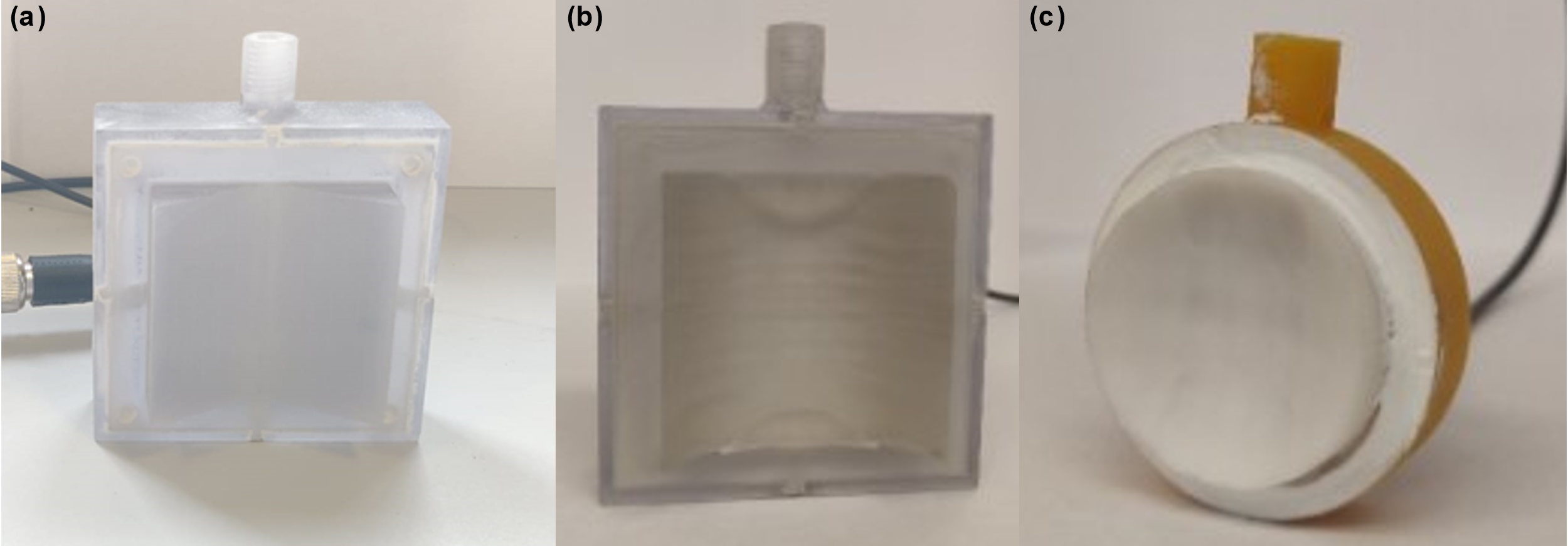


Figure S3. The fabrication of the transducers used to create the axial pressure planes. At 1.5 MHz, and a 4.5 square piezoceramic element, transducer 1(a) has a geometric lens shaped as a negative prism, and transducer 2 (b) has a holographic lens. Transducer 3 (c) is made from a 2 MHz, 3.5-cm circular piezoceramic element and a holographic lens.

# Holography scan

The three dimensional pressure field was reconstructed using the hologram propagation to show pressure field from the source forward along the acoustic axis. Each row of Figure S4 presents the source phase, the measured imposed pressure amplitude in the *xy*-plane, and axial pressure amplitude in *zx­*-plane at *y* = 0. The right column of Figure S4 shows the axial pressure from the source to beyond the image plane where the region of formation of the parallel pressure planes is bounded by the white rectangle.


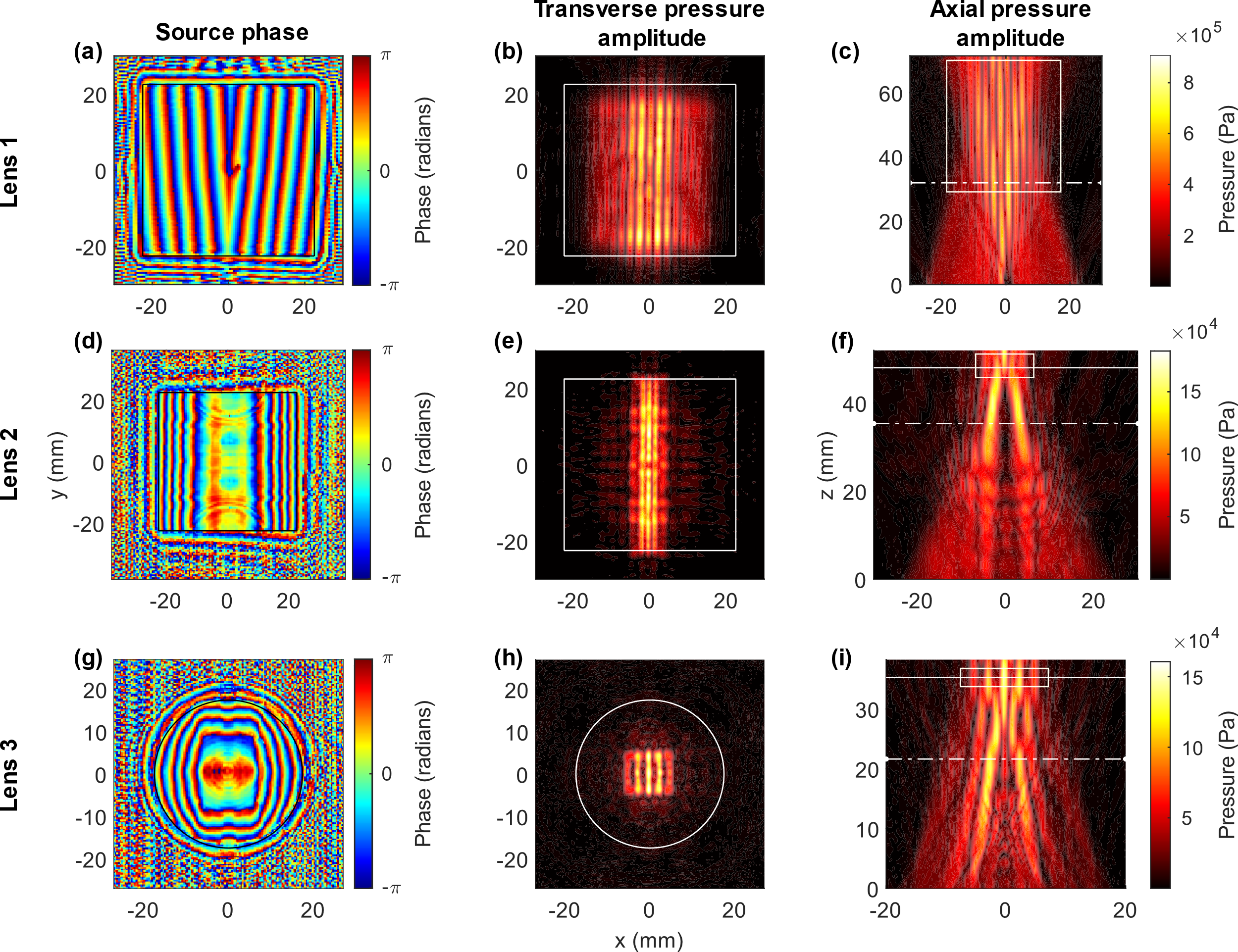


Figure S4. Results of holography scan. Each row represents the result from lens 1 (top), 2 (middle) and 3 (bottom). The columns are source phase (left), measured image of pressure amplitude in the transverse *xy*-plane (middle), and axial *zx*-plane (right). In the right column plots, the solid white box shows the axial extension length of the parralel planes of pressure amplitude, the solid white line marks the location of the image plane shown in the middle column, and the dashed white line marks the location of the holography scan plane. The field produced from lens 1 is equivalent to that of two sources with their acoustic axes seperated by an angle 2*θ* which outputs approximately a standing wave in the transverse direction. Lens 2 and 3 produced five parallel axial planes as predicted by simuation (see Figure S3). For lens 2, the outer planes are weakly formed and never acheive full illuination, while lens 3 produced five planes with outer planes slightly weaker than predicted by simulation.

# Sensitivity analysis of holographic lenses made with IASA

The sensitivity of the pressure image obtained from lens 2 and 3 was investigated to the source phase and amplitude by testing two virtual sources using different boundary condition cases for each lens. The virtual sources’ boundary conditions were 1) simulation amplitude and measured phase *A*_s_$\Phi_{m}$ and 2) measured amplitude and simulation phase *A*_m_$\Phi_{s}$. FSI was used to score the agreement between these sources to simulation (Figure S5d and h) in order to qualitatively evaluate the source phase and amplitude on the pressure distribution in the desired imposed image plane. These FSI score of the virtual sources were compared with those obtained from the experimental measurement (Figure S4e and h) to assess the sensitivity to each boundary condition.

Similarly the sensitivity of the results was quantified using the same virtual source analysis for different lens’s surface morphology. A version of lens 3 was fabricated and had its acoustic field measured using holography. The lens was fabricated from a wrapped source phase and had its results scored with FSI and compared to those obtained from an unwrapped lens.

Figure S5 presents results from both virtual sources, *VS*(*A*_m_$\Phi_{s}$) and *VS*(*A*_s_$\Phi_{m}$) for both holographic lenses 2 and 3. Propagation from *VS*(*A*_m_$\Phi_{s}$) resulted in full formation of all 5 planes with certain regions with relatively high intensity, Figure S5b and f. Results from *VS*(*A*_s_$\Phi_{m}$) exhibited attenuated pressure output toward the edges of the pressure image (Figure S5c and g). Consequently for lens 2, the FSI improved from 0.939 to 0.941 and 0.961 for *VS*(*A*_s_$\Phi_{m}$) and *VS*(*A*_m_$\Phi_{s}$); and for lens 3, FSI improved from 0.953 to 0.96 and 0.982, respectively(see Table S1). The results indicate that the errors in the phase boundary condition produce greater discrepancy between the desired and measured pressure because when the simulation source phase is used a higher FSI score is achieved than that resulting from when the simulation amplitude is used.

Table S1. Comparison of FSI for different source boundary condition cases.

| Source boundary condition | Experimental (non-virtual source)  (see paper) | Simulation Amplitude and measured phase *VS*(*A*_s_$\Phi_{m}$) | Measured amplitude and simulation phase *VS*(*A*_m_$\Phi_{s}$) |
| --- | --- | --- | --- |
| Lens 2 | 0.939 | 0.941 | 0.961 |
| Lens 3 | 0.953 | 0.96 | 0.982 |


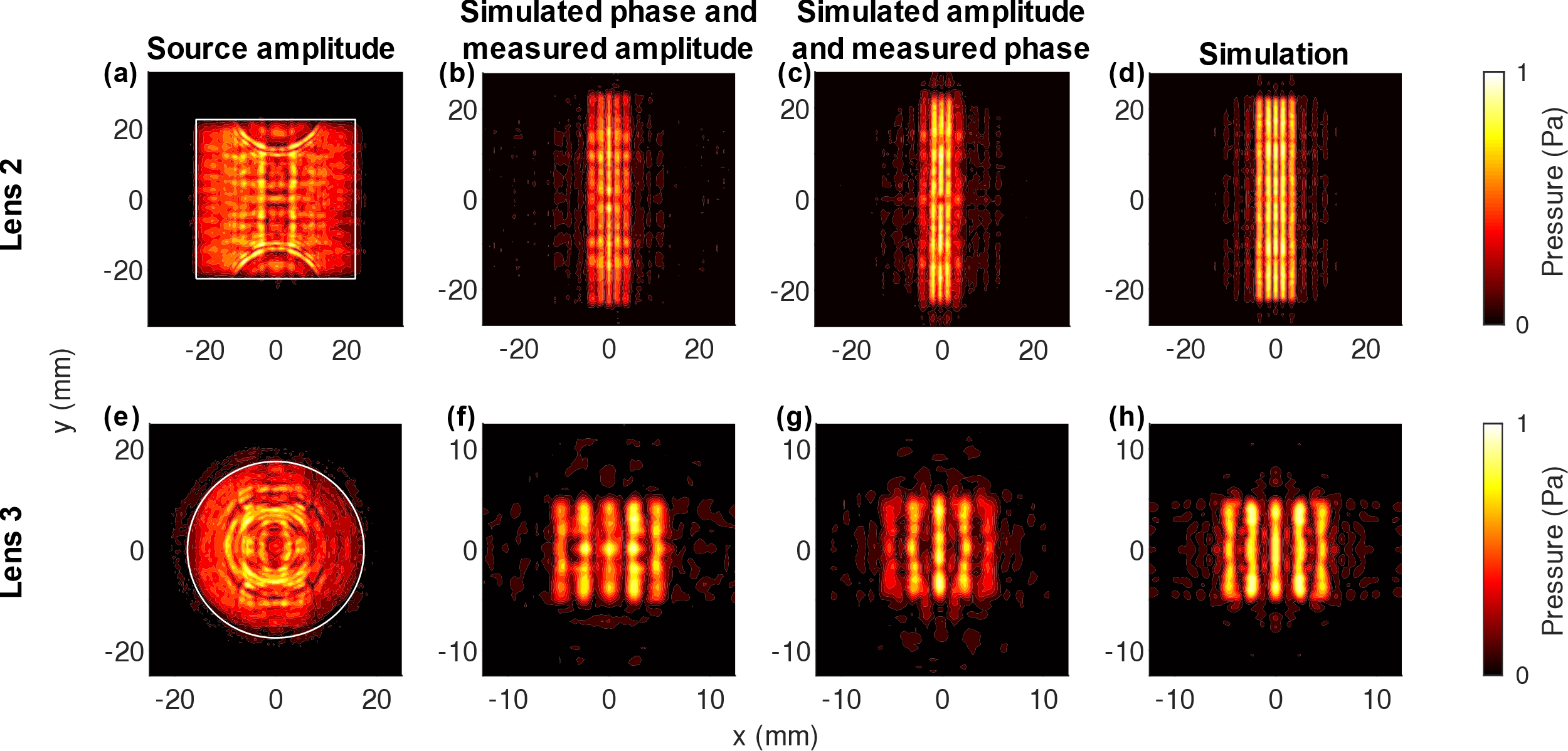


Figure S5. Sensistivity of the pressure results to the source boundary conditions. The measured source amplitude of lens 2 and 3 are nonuniform with attenuated regions that correpsonds to thick lens material (left). Results of virtual source using simulated phase and measured amplitude boundary conditions (b and f), using simulated amplitude and measured phase boundary conditions (c and g) are compared with simulation results (d and h), and shown for lens 2 (top) and 3 (bottom). The results present the sensitivity of the holograms to both the amplitude and phase source variations.

The new iteration of lens 3 made from a wrapped source phase was thinner and had discontinuities ^4^, thus, allowing us to evaluate the effects of lens material attenuation in addition to the smooth continuous surface morphology of an unwrapped phase lens. The new iteration of lens 3 had a maximum thickness of (1/*λ*_m_ – 1/*λ*_l_)^-1^ = 1.8 mm (see Figure S6d), where *λ*_m_ and *λ*_l_ are the wavelength in water and lens material. The holography measurements showed that the pressure distribution was less accurate than the results from an unwrapped lens with an FSI of 0.928 vs. 0.953 (Figure S6a vs. Figure S4h). Figure S6 shows that the results from the wrapped lens were also more sensitive to the experimental phase than the amplitude (Figure S6b & c) as the FSI changed from 0.928 to 0.926 and 0. 989 for *VS*(*A*_s_$\Phi_{m}$) and *VS*(*A*_m_$\Phi_{s}$), respectively. Table S2 summarizes the results of the FSI score for each phase morphology used.

Table S2. Unwrapped vs. wrapped phase lens 3 effects on measured pressure.

| Source boundary condition | Experimental (non-virtual source) | Simulation Amplitude and measured phase *VS*(*A*_s_$\Phi_{m}$) | Measured amplitude and simulation phase *VS*(*A*_m_$\Phi_{s}$) |
| --- | --- | --- | --- |
| Unwrapped phase | 0.953 | 0.960 | 0.982 |
| Wrapped phase | 0.928 | 0.926 | 0.989 |

Phase wrapping worsened the accuracy of the results the FSI for *VS*(*A*_s_$\Phi_{m}$) was 0.926 vs. 0.960 of the unwrapped phase (see Table S2). Further analysis of phase wrapping shows that the fabricated lens introduces substantial deviations to the acoustic output as the lower regions of the lens are shadowed by the higher edges that block the acoustic rays, thus, adding perturbations to the acoustic field. The measured source phase was used to get the lens profile, which confirmed that perturbation occurring from lens stepping resulted in a substantially different lens profile than the fabricated profile (Figure S6d). The effect of lens attenuation was reduced as the FSI for *VS*(*A*_m_$\Phi_{s}$) obtained from the unwrapped lens was improved from 0.982 to 0.989 for the phase wrapped lens (see Table S2). This agrees with our results in Figure S5 that illustrate the experimental source amplitude to be nonuniform, contradictory to what was imposed by IASA, and with lower output due to higher attenuation caused by the thicker regions of the holographic lenses. Thus, although phase unwrapping causes higher attenuation, it produces the most accurate phase boundary condition and final obtained pressure image.


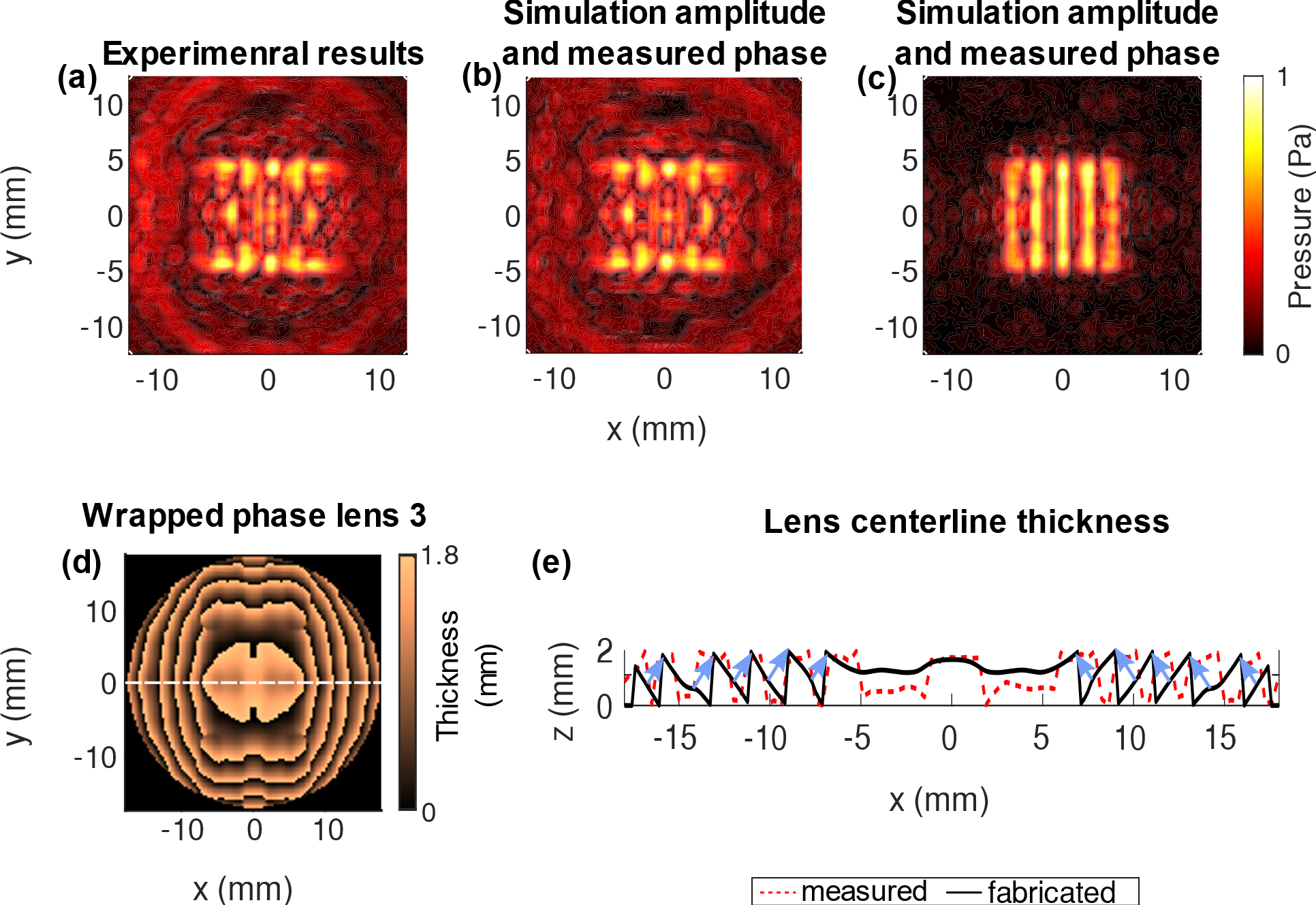


Figure S6. Analysis of wrapped-phase lens 3 output and sensitivity of the pressure results to the source boundary conditions. The measured image pressure amplitude (a) deviates from the desired imposed image of Fig. S2d. Results of virtual source using measured phase and simulation amplitude *VS*(*A*_s_$\Phi_{m}$) (b), and using measured amplitude and simulation phase *VS*(*A*_m_$\Phi_{s}$) (c) boundary conditions. The results indicates that holograms are more sensitive to phase variations than amplitude. Sectional cuts (e) of the fabricated wrapped lens surface (solid) and effective lens surface from holography measurements (dashed line) along the *x*-axis (white dashed line in d) show the shadowing effect on the troughs of the lens’ surface causes large pertubations of the source output phase.

# Acoustic radiation forces

Gor’kov potential is defined to be:

$$U=\pi r^{3}\rho\left[ \frac{p_{in}^{2}}{3\rho^{2}c^{2}}f_{1}-\frac{v_{in}^{2}}{2}f_{2} \right]$$

For harmonic wave the velocity is:

| $\vec{v}=\frac{\nabla P}{i\omega\rho}$ and $\left\vert v \right\vert^{2}=\frac{1}{\omega^{2}\rho^{2}}\nabla P\cdot\nabla P^{*}$ | **[S4]** |
| --- | --- |

where Z^*^ is the conjugate of Z.

For ease of calculation, we will denote *U* to be:

$$U=A\left| P \right|^{2}+B\left| v \right|^{2}$$

where $B= \frac{\tilde{B}}{\omega^{2}\rho^{2}}$, and *A* and $\tilde{B}$ are found from the coefficients of *U*.

From Gor’kov potential and equation [S4]:

$$U=AP\cdot P^{*}+B\nabla P\cdot\nabla P^{*}$$

and forces is defined to be:

$F=-\nabla U$.

Then,

$\nabla U= e_{\mu}\frac{\partial U}{\partial x_{\mu}}$, $e_{\mu}$ is a unit vector, and $\mu=1, 2, 3$.

The second term,

$\nabla P\cdot\nabla P^{*}=e_{\mu}\frac{\partial P}{\partial x_{\mu}}\cdot e_{\gamma}\frac{\partial P^{*}}{\partial x_{\gamma}}$, where $e_{\mu}\cdot e_{\gamma}=\delta_{\mu\gamma}$ is Kronecker delta,

then,

$\nabla U=Ae_{\mu}\frac{\partial}{\partial x_{\mu}}\left( P\cdot P^{*} \right)+Be_{\mu}\frac{\partial}{\partial x_{\mu}}\left( \frac{\partial P}{\partial x_{\gamma}}\frac{\partial P^{*}}{\partial x_{\gamma}} \right)$.

Therefore,

$\nabla U=2Ae_{\mu}\cdot Re\left( \frac{\partial P}{\partial x_{\mu}}P^{*} \right)+2Be_{\mu}\cdot Re\left( \frac{\partial^{2}P}{\partial x_{\mu}\partial x_{\gamma}}\frac{\partial P^{*}}{\partial x_{\gamma}} \right)$.

Solving for *A* and *B*  coefficients using *U,* the acoustic radiation force in the *μ* direction becomes:

$$F_{\mu}=\frac{\pi r^{3}}{\rho c^{2}}\cdot Re \left[ -\frac{2f_{1}}{3}\cdot\frac{\partial P}{\partial x_{\mu}}P^{*}+\frac{c^{2}f_{2}}{\omega^{2}}\cdot\frac{\partial^{2}P}{\partial x_{\mu}\partial x_{\gamma}}\frac{\partial P^{*}}{\partial x_{\gamma}} \right]$$

The solution for *F* three-dimensional space can be found using the angular spectrum definition shown below:

$$p\left( x,y,z \right)=\frac{1}{4\pi^{2}}\iint S\left( k_{x},k_{y} \right)e^{ik_{x}x+ik_{y}y+ik_{z}z} dk_{x}{dk}_{y}$$

where $k_{z}=\sqrt{k^{2}-k_{x}^{2}-k_{y}^{2}}$.

# Radiation force alignment

The supplemental movies S1-S3 show the alignment of the microspheres after ultrasound exposure from all three lenses. The microspheres are illuminated with a 1.2-mm thick laser sheet produced by a 532-nm laser diode. Figure S7 and Movie S4 show the alignment of the microspheres from lens 3 where a high concentration of microspheres was used so that the unstable trapping regions of the field or anti-nodes were dark spaces as the microspheres were pushed away. The high concentration of microspheres resulted in a reduction of light scattering from the spheres and absence of light where no trapping was present allowing the visualization of the full particle agglomeration along the secondary trapping regions as well as within saddle regions of the field.

| 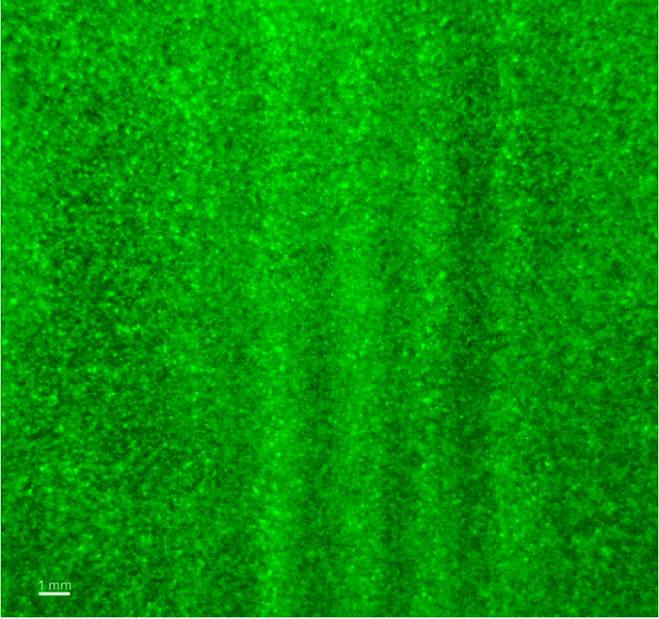 | 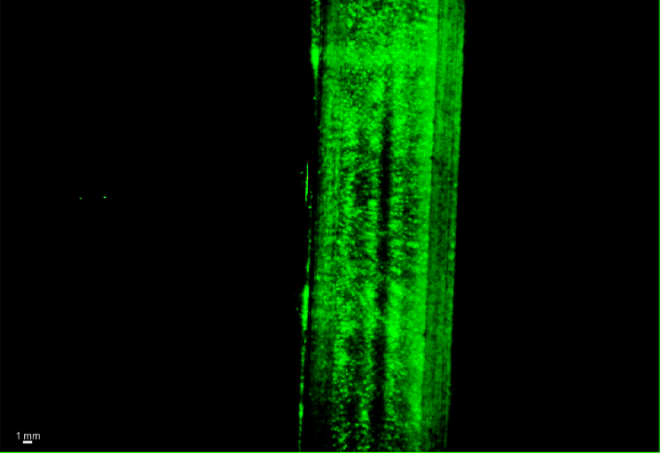 |
| --- | --- |
| Movie S1. Alignment of microshperes using holographic lens 1 at 1.5 MHz. The microshperes agglomerate along seprate vertical planes as visualized by a camera looking directly at the the cuvette. The scale bar is 1 mm in length. | Movie S2. Alignment of microshperes using holgoraphic lens 2 at 1.5 MHz. The microshperes agglomerate along seprate 4 vertical planes as visualized by a camera looking directly at the the cuvette. The scale bar is 1 mm in length. |


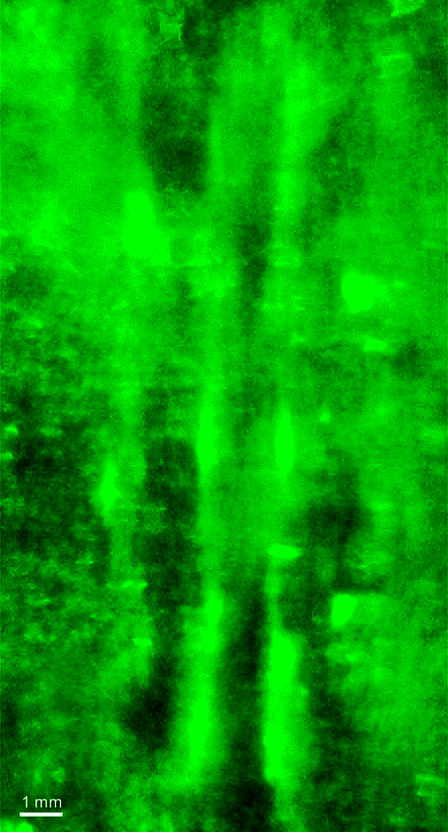


Movie S3. Alignment of microshperes using holgoraphic lens 3 at 2 MHz. The microshperes agglomerate along seprate 4 vertical planes as visualized by a camera looking directly at the the cuvette. The fourth line is weekly formed on the outside right. The scale bar is 1 mm in length.


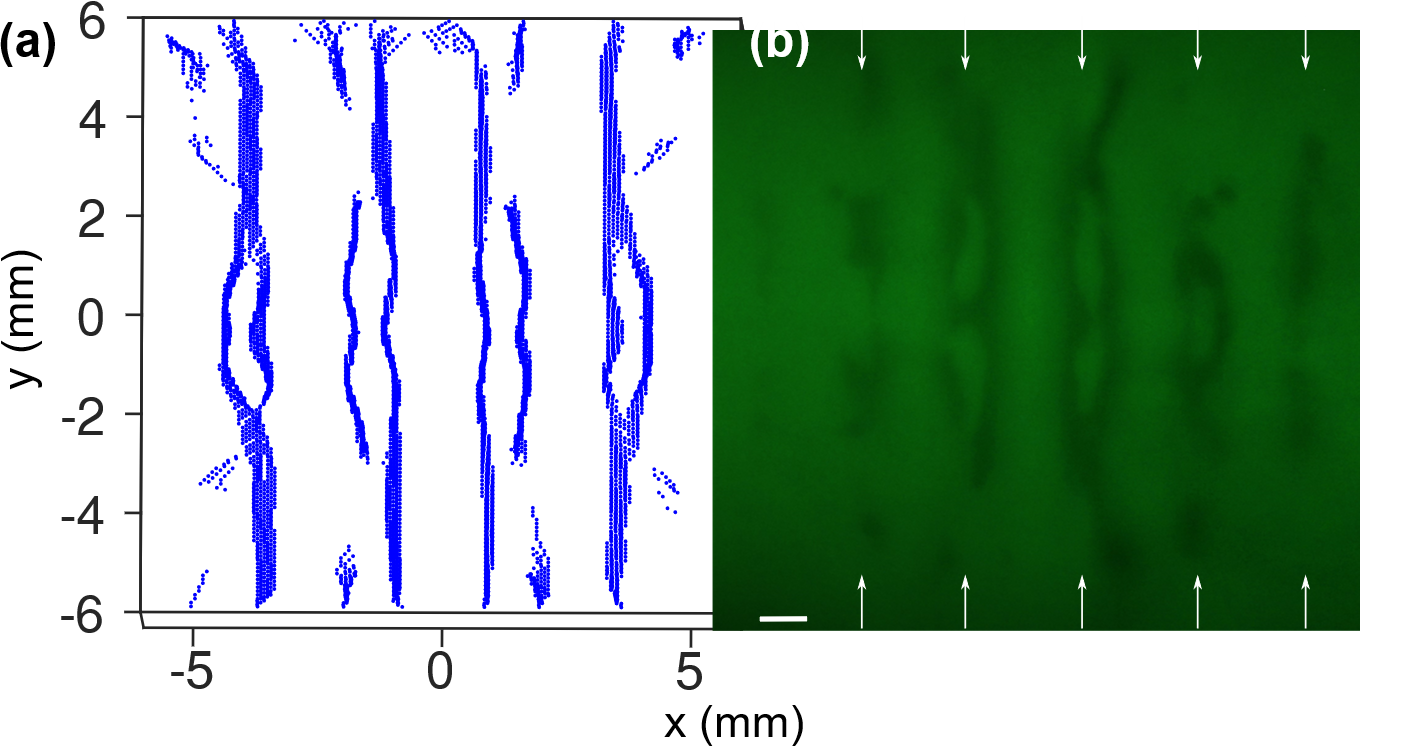


Figure S7. Alignment of microshperes using holgoraphic lens 3 at 2 MHz. Simulation of the partcle patterning in three-dimensional at the plane of interest (a) is compared to measurements (b). A negative image was created by using a high concnetaration of micrhospheres where fully dark region with complete absense of light are areas where no particle trapping occurs. The purpose of this image that it shows the particle agglormeration in regions of saddle points, and secondary trapping lines that occrus between the four main vertical planes. The scale bar in (b) is 1 mm in length.


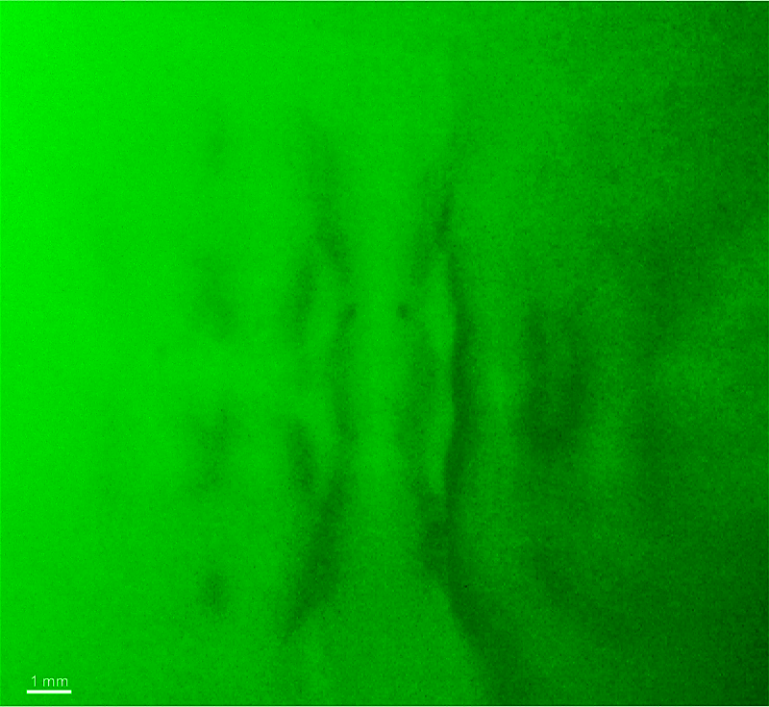


Movie S4. Alignment of microshperes using holgoraphic lens 3 at 2 MHz. A negative image was created by using a larger amount of microsphere solution in the cuvette, where fully dark region with complete absense of light are areas where no particle trapping occurs. The microshperes agglomerate along seprate 4 vertical planes as visualized by a camera looking directly at the the cuvette. The fourth line is weekly formed on the outside right. The movie captures the particle agglormeration in regions of saddle points, and secondary trapping lines that occrus between the four main vertical planes. The scale bar is 1 mm in length.

# Reference

1. Sapozhnikov, O. A. & Bailey, M. R. Radiation force of an arbitrary acoustic beam on an elastic sphere in a fluid. *The Journal of the Acoustical Society of America* vol. 133 661–676 at https://doi.org/10.1121/1.4773924 (2013).

2. Melde, K., Mark, A. G., Qiu, T. & Fischer, P. Holograms for acoustics. *Nature* **537**, 518–522 (2016).

3. Zhao, Z. *et al.* Robust 2D phase unwrapping algorithm based on the transport of intensity equation. *Meas. Sci. Technol.* **30**, 015201 (2019).

4. Hadimioglu, B. *et al.* High-efficiency Fresnel acoustic lenses. in *1993 Proceedings IEEE Ultrasonics Symposium* vol. 146 579–582 vol.1 (IEEE, 1993).
